# Association between plant-based dietary patterns and dementia among Chinese older adults

**DOI:** 10.3389/fnut.2025.1669647

**Published:** 2025-11-21

**Authors:** Xiaobing Xian, Yue Chen, Yandi Fu, Ziyi Chen, Jie Xiang, Ze Han, Li Zeng, Jiaxia Li, Yuanyuan Wang, Kun Shen

**Affiliations:** 1 The Thirteenth People's Hospital of Chongqing, Chongqing, China; 2 Chongqing Geriatrics Hospital, Chongqing, China; 3 The First Clinical College, Chongqing Medical University, Chongqing, China; 4 School of Pediatric, Chongqing Medical University, Chongqing, China; 5 College of Traditional Chinese Medicine, Chongqing Medical University, Chongqing, China; 6 The Second Clinical College, Chongqing Medical University, Chongqing, China; 7 School of Stomatology, Chongqing Medical University, Chongqing, China; 8 School of Mathematics and Statistics, Chongqing Technology and Business University, Chongqing, China

**Keywords:** plant-based dietary patterns, dementia, older adults, CLHLS, external validation

## Abstract

**Background:**

Dementia has become a major concern with the rapid aging of the population. While plant-based dietary habits are widely regarded as beneficial, evidence on their associations with dementia among Chinese older adults is still limited.

**Methods:**

Our study included 9,360 individuals from the 2018 data collection of the Chinese Longitudinal Healthy Longevity Survey (CLHLS), a nationally representative, cross-sectional survey. We developed three plant-based diet indices—the overall (PDI), healthful (hPDI), and unhealthful (uPDI)—using a simplified food frequency questionnaire. Multivariable logistic regression, adjusted for various covariates, was used to examine the associations between these diet indices and dementia prevalence. Additional analyses encompassed restricted cubic splines (RCS), subgroup analyses, interaction tests, and sensitivity analyses to assess the robustness of the findings. We further validated the results in an independent group of 588 older adults from Chongqing, China.

**Results:**

The observed prevalence of dementia among older adults in the CLHLS sample was 10.67%, while a cross-sectional survey conducted in Chongqing reported a prevalence of 13.78%. Results from logistic regression models controlling for all covariates indicated that the PDI, hPDI, and uPDI were significantly associated with dementia among Chinese older adults (PDI: OR = 0.964, 95% CI = 0.951–0.977; hPDI: OR = 0.976, 95% CI = 0.963–0.990; uPDI: OR = 1.012, 95% CI = 1.001–1.024). The restricted cubic spline (RCS) analysis further confirmed a significant dose-response relationship between PDI, hPDI, and dementia. Interaction analysis revealed that diabetes and physical exercise significantly modified these associations. Validation results based on the older adult population in Chongqing were consistent with the above findings.

**Conclusion:**

Plant-based dietary patterns showed significant cross-sectional associations with dementia prevalence among Chinese older adults. The modifying effects of diabetes and exercise highlight the importance of considering individual characteristics when examining diet–dementia relationships.

## 1 Introduction

Dementia, a neurodegenerative disorder, is increasingly recognized as a global public health challenge, largely attributable to the aging population. It is defined by a progressive deterioration across at least two cognitive areas—such as memory, language, executive function, and visuospatial abilities—often accompanied by shifts in personality and disruptive behaviors, thereby significantly hindering an individual's capacity for independent daily living and social engagement (1, 2). The 2021 World Alzheimer Report indicated an estimated 57 million individuals living with dementia globally in 2021, a figure anticipated to reach ~78 million by 2030 and a staggering 139 million by 2050 (3, 4). In China, dementia impacts roughly a quarter of the world's total dementia population, imposing a substantial strain on policymakers, healthcare professionals, and families alike (5). Modern geriatrics emphasizes disease prevention, identifying modifiable factors is important for the early identification and management of dementia.

The 2024 Lancet Standing Commission estimated a combined weighted population attributable fraction (PAF) of ~45% for 14 modifiable risk factors, including low education, hearing loss, hypertension, smoking, obesity, depression, physical inactivity, diabetes, excessive alcohol use, traumatic brain injury, air pollution, social isolation, high LDL-cholesterol (midlife), and untreated vision loss (late life), indicating that, theoretically, nearly half of dementia cases might be prevented or delayed under optimal risk-factor control (6). Although dietary patterns are not explicitly included among the 14 modifiable factors, they constitute an important lifestyle behavior closely linked to several of these risks and have increasingly attracted attention for their broad influence on chronic disease outcomes. Exploring such dietary patterns offers significant advantages over investigating single nutrients. While single-nutrient studies often overlook the synergistic effects among foods and the comprehensive impact of overall dietary structure on health, dietary patterns offers a more comprehensive reflection of an individual's eating habits and their long-term health effects.

Among various dietary patterns, plant-based diets have garnered growing international interest. Broadly defined, they emphasize foods of plant origin while restricting or completely excluding animal-derived products. This category ranges from strict vegan diets to less restrictive forms such as lacto-ovo vegetarian, pescatarian, or semi-vegetarian diets, and also extends to health-oriented patterns like the Mediterranean and DASH diets, which still include limited amounts of animal foods but place strong emphasis on plant-based sources. The unifying feature across these approaches is the prioritization of abundant plant-derived foods (7). Plant foods are rich in dietary fiber, vitamins, minerals, and polyphenols, which may exert beneficial effects through antioxidant and anti-inflammatory pathways, as well as by modulating the gut microbiota. Accordingly, they have been linked to potential advantages in cardiovascular health, type 2 diabetes, certain cancers, and the preservation of cognitive function. Evidence from international studies further indicates that plant-based diets are closely associated with reduced risks of cardiovascular disease, type 2 diabetes, and cancer, as well as with better cognitive outcomes (8, 9). In addition, a substantial body of literature has shown that dietary patterns such as the Mediterranean, DASH, and MIND diets are related to lower prevalence of cardiovascular disease, diabetes, and cognitive decline (10–12). However, these dietary patterns have primarily been developed in Western populations, whose eating habits differ considerably from those of older adults in China.

Traditionally, Chinese dietary habits have been characterized by a strong reliance on plant-based foods, with cereals and vegetables serving as staples. Carbohydrates constitute the main source of energy, while protein intake has largely been derived from grains and legumes (13). However, with rapid economic development and accelerated urbanization, this predominantly plant-centered dietary pattern has undergone marked transitions. The most notable shifts include rising consumption of animal-source foods and edible oils, accompanied by a relative decline in cereals, whole grains, and certain types of vegetables (14–16). These changes in the balance between plant- and animal-based foods have been linked not only to the increasing prevalence of obesity, cardiovascular disease, and type 2 diabetes, but may also have important implications for cognitive health in the elderly.

Although plant-based diets have been theoretically and empirically linked to various health benefits in international studies, direct evidence on their associations with dementia remains limited. Especially among older adults in China, previous research has primarily focused on examining the associations between dietary quality or diversity and cognitive impairment (17, 18). Mild cognitive impairment, however, is generally considered a prodromal stage of dementia (19), and thus studies directly examining plant-based dietary patterns and dementia are lacking. One study conducted in the Rotterdam cohort suggested a potential link between plant-based diets and dementia risk (20), yet its participants were predominantly European, with dietary practices and genetic backgrounds that differ considerably from those of Chinese populations. To date, no large-scale study has systematically examined these associations in Chinese older adults.

Addressing this gap, the present study used data from the Chinese Longitudinal Healthy Longevity Survey (CLHLS) together with an external dataset from older adults in Chongqing to evaluate the associations of plant-based diet indices (PDI, hPDI, and uPDI) with dementia, and to explore potential effect modifiers. This study aims to provide evidence on the relationship between plant-based diets and dementia in the Chinese elderly population, contributing knowledge that may inform strategies to improve quality of life in later years.

## 2 Materials and methods

### 2.1 Study participants

The data utilized in the present inquiry were sourced from the Chinese Longitudinal Healthy Longevity Survey (CLHLS), a prospective, nationally representative cohort investigation managed by the Center for Healthy Aging and Development Studies, Peking University/National Institute for Development Studies. The extensive survey covers 23 provincial-level administrative regions across China, concentrating on residents who are 65 years of age or older. Information gathered included fundamental demographic attributes, socioeconomic markers, evaluations of functional ability, and aspects related to lifestyle behaviors (21). Ethical approval for this investigation was granted by Peking University's Institutional Review Board (IRB00001052 – 13074), and all individuals participating provided written informed consent.

**Figure 1:**
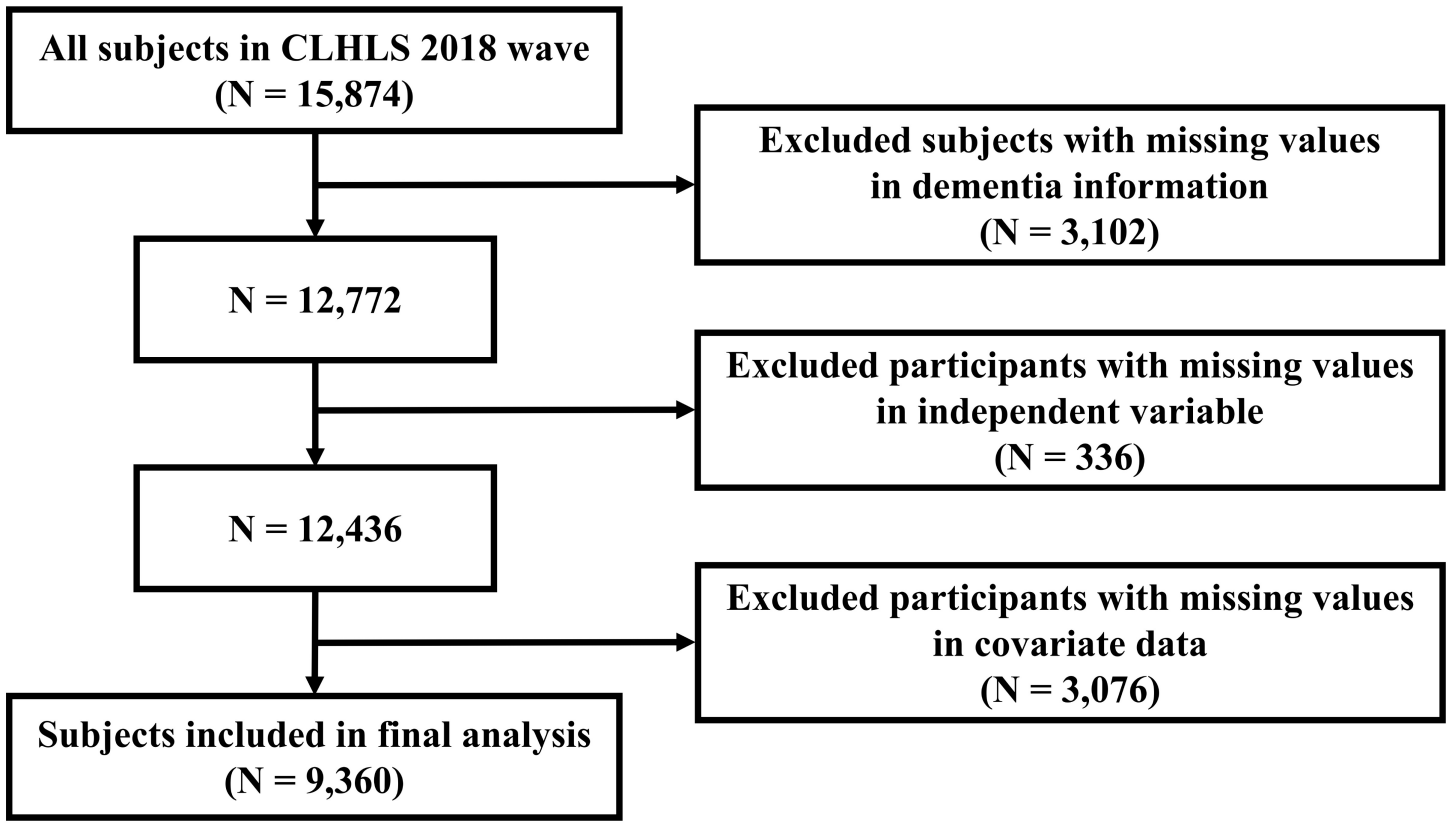
Flowchart for the study design and participants.

We employed cross-sectional data from the 2018 wave of the CLHLS, comprising an initial cohort of 15,874 participants. After excluding 3,102 individuals with missing dementia-related data and subsequently removing cases with incomplete independent variable or covariate information, the ultimate analytical cohort included 9,360 individuals. A comprehensive depiction of the data preparation methodology is presented in Figure 1.

For additional corroboration of the link between plant-based eating habits and dementia among older adults, a cross-sectional validation investigation was conducted in Chongqing, China. This research included 588 community-resident individuals, 65 years of age or more, who attended the Thirteenth People's Hospital of Chongqing (Chongqing Geriatric Hospital) during June and July 2025. According to the patients' medical records, all participants were recruited from various community neighborhoods in the main urban area of Chongqing. Dementia diagnoses adhered to standardized criteria consistent with those employed in the CLHLS. Certified research staff administered standardized questionnaires through in-person interviews. The study's methodology conformed to the Declaration of Helsinki guidelines and secured ethical clearance from the Institutional Review Board of the Thirteenth People's Hospital of Chongqing (Approval No. 2025-022-01). No participants were enrolled without obtaining written informed consent following full disclosure of study objectives and methodology.

### 2.2 Plant-based diet indices

In accordance with the approach proposed by Satija et al. (22, 23), we constructed three dietary indices—the overall Plant-based Diet Index (PDI), the healthful Plant-based Diet Index (hPDI), and the unhealthful Plant-based Diet Index (uPDI)—to evaluate plant-based dietary patterns among Chinese older adults. Dietary information was obtained from the simplified food frequency questionnaire (FFQ) in the Chinese Longitudinal Healthy Longevity Survey (CLHLS). The FFQ has been validated in multiple CLHLS-based studies and demonstrated high feasibility and scientific reliability (24–26). It covers 16 major food groups that represent commonly consumed foods in the Chinese diet. Participants reported the frequency of their consumption of various foods (“Almost every day,” “≥1 time/week,” “≥1 time/month,” “Occasionally,” or “Rarely or never”), while for whole grains, vegetable oil, refined grains, and animal fat, they reported whether or not they consumed these items. Based on both food origin and nutritional characteristics, all food items were first divided into two main categories: plant-based and animal-based foods. Plant-based foods included whole grains, nuts, tea, legumes, garlic, vegetable oils, fresh fruits, fresh vegetables, pickled vegetables, refined grains, and sugar, while animal-based foods included fish and seafood, meat, animal fats, eggs, and dairy products (27, 28). Building upon this, plant-based foods were further categorized into two distinct groups based on their nutritional content and established health outcomes: healthy plant-based foods (whole grains, fruits, vegetables, vegetable oils, legumes, garlic, nuts, and tea) and unhealthy plant-based foods (refined grains, sugar, and pickled vegetables) (17, 22).

Individuals indicated their consumption frequency for every food item via a five-point grading system, ranging from 1 (seldom/never) to 5 (most regularly consumed). Our investigation derived three distinct index scores: the Overall Plant-based Diet Index (PDI), the Healthy Plant-based Diet Index (hPDI), and the Unhealthy Plant-based Diet Index (uPDI). The scoring methodology varied slightly among these three indices. For the PDI, all plant-based items received positive scores (ranging from 1 to 5 points) irrespective of their healthfulness, while animal-derived foods were given inverse scores (5–1 points). The hPDI allocated favorable scores (1–5 points) to healthy plant-based options, but inverse scores (5–1 points) to both less healthy plant foods and all animal-based foods. In contrast, the uPDI conferred positive scores (1–5 points) to unhealthy plant-based selections, assigning inverse scores (5–1 points) to both healthy plant-based and animal-based foods (26). The calculation methods for the three indices and food classification criteria are detailed in Supplementary Table S1.

### 2.3 Dementia

Consistent with the Diagnostic and Statistical Manual of Mental Disorders (DSM-5) and the International Classification of Diseases, 10th Revision (ICD-10), a diagnosis of dementia necessitates the co-occurrence of cognitive and functional impairments, or a physician's confirmed diagnosis of dementia or related memory disorders. This working definition has received empirical substantiation from extensive population-based investigations (29–31).

Cognitive impairment was evaluated employing the Chinese version of the Modified Mini-Mental State Examination (CMMSE). This scale comprises 24 items across 7 cognitive function categories, including general competence, reactivity, attention and numeracy, recollection skills, language, comprehension, and self coordination skills. Detailed item descriptions and scoring criteria are provided in Supplementary Table S2. The CMMSE's overall score spans from 0 to 30, where diminished values signify poorer cognitive abilities. This scale has been extensively employed in epidemiological research for cognitive screening and demonstrates strong validity within Chinese demographic cohorts. In the CLHLS, the CMMSE was administered by trained interviewers through face-to-face interviews, and CMMSE assessments were conducted at baseline and during each follow-up survey. Assessment results were grouped by education level: Among older individuals, those lacking formal education and achieving a score of 16 or below, or participants with 1–6 years of schooling scoring 19 or less, or individuals with over 6 years of education scoring 23 or under, were deemed to exhibit cognitive decline; while all other instances were classified as having typical cognitive performance (32, 33).

Functional capabilities were assessed using the Katz Activities of Daily Living (ADL) scale, obtained from the CLHLS (34, 35). Participants were classified as exhibiting functional limitations if they demonstrated dependence on external assistance for performing one or more essential activities of daily living (encompassing personal hygiene, mobility, dressing, elimination, feeding, and continence management).

Self-reported physician-diagnosed dementia was based on the question “Have you now been diagnosed with dementia by a hospital?” Participants or their caregivers were considered to have dementia if they answered “Yes” to this question.

### 2.4 Covariates

To control for potential confounding factors that might interfere with the study results, this study included 15 covariates from four dimensions: demographic characteristics, socioeconomic status, lifestyle and health status. These included age, gender, residence, marital status, economic status, education level, smoking, drinking, exercise, living arrangements, hypertension, diabetes, heart disease, dyslipidemia, and body mass index (BMI). The specific measurement methods and assignment rules for each variable are detailed in Supplementary Table S3.

### 2.5 Statistical analysis

In both datasets, categorical variables were expressed by frequencies and percentages [*n*(%)], and Chi-square tests were employed to compare the prevalence of dementia across various features. To further examine the associations of PDI, hPDI, and uPDI with dementia, three separate logistic regression models were constructed under the condition that the minimum sample size requirements based on the events-per-variable (EPV) principle were satisfied. Model 1 did not include adjustments for any covariates; Model 2 incorporated adjustments for gender, age, residence, marital status, economic status and education level; Model 3 additionally adjusted for smoking, drinking, living arrangements, BMI, hypertension, diabetes, heart disease and dyslipidemia. A variance inflation factor (VIF) < 10 was considered to indicate no multicollinearity issues among variables. The E-value metric was employed to quantify the potential influence of unmeasured confounders on the observed associations. Subsequently, restricted cubic splines (RCS) were utilized to investigate potential non-linear dose-response relationships between plant-based diet indices (PDI, hPDI, uPDI) and dementia outcomes. Stratified analyses and interaction testing were performed to assess the consistency of these associations across population subgroups and to detect potential effect modifiers. To mitigate multiple comparison concerns and control the false positive rate, we implemented False Discovery Rate (FDR) correction for all subgroup analysis *p*-values (36).

To further test the robustness of our conclusions, we conducted three sensitivity analyses. First, to address potential bias arising from complete-case analysis, we implemented the Multiple Imputation by Chained Equations (MICE) algorithm to handle missing data. Second, we systematically excluded individuals with major cardiometabolic comorbidities (hypertension, diabetes mellitus, cardiovascular disease, and dyslipidemia), as these clinical populations often adhere to therapeutic dietary modifications. Third, given that dementia generally has a relatively low prevalence, odds ratios from logistic regression may overstate the strength of associations. Therefore, a Poisson regression model with robust standard errors (Huber-White sandwich estimator) (37) was additionally applied to re-estimate the prevalence ratios for secondary evaluation of the associations.

Statistical analyses were performed utilizing R version 4.3.0, with a two-tailed *p*-value below 0.05 set as the threshold for statistical significance.

## 3 Results

### 3.1 Characteristics of study participants

The analytical cohort consisted of 9,360 participants, among whom dementia prevalence was recorded in 999 cases (10.67%), while 8,361 individuals (89.33%) were non-demented, as detailed in Table 1. Among them, 55.06% were male and 44.94% were female. The majority of participants were aged 80 years or older (61.77%). The proportions of participants in the dementia and non-dementia groups differed significantly across various age, gender, marital status, economic status, education level, smoking status, drinking status, exercise habits, living arrangements, BMI, hypertension, diabetes, and dyslipidemia categories (*P* < 0.05).

**Table 1:** Characteristics of the study participants at baseline by dementia.

**Variables**	**Total (*n* = 9,360)**	**Without dementia (*n* = 8,361)**	**Dementia (*n* = 999)**	**Statistic**	***P***
**Age**, ***n*** **(%)**					
< 80	3,578 (38.23)	3,553 (99.30)	25 (0.70)	χ^2^ = 604.42	< 0.001
≥80	5,782 (61.77)	4,808 (83.15)	974 (16.85)		
**Gender**, ***n*** **(%)**					
Female	5,154 (55.06)	4,445 (86.24)	709 (13.76)	χ^2^ = 114.36	< 0.001
Male	4,206 (44.94)	3,916 (93.11)	290 (6.89)		
**Residence**, ***n*** **(%)**					
Rural	3,939 (42.08)	3,518 (89.31)	421 (10.69)	χ^2^ = 0.00	0.968
Urban	5,421 (57.92)	4,843 (89.34)	578 (10.66)		
**Marital status**, ***n*** **(%)**					
Married	5,175 (55.29)	4,289 (82.88)	886 (17.12)	χ^2^ = 504.69	< 0.001
Other	4,185 (44.71)	4,072 (97.30)	113 (2.70)		
**Economic status**, ***n*** **(%)**					
Good	1,826 (19.51)	1,690 (92.55)	136 (7.45)	χ^2^ = 37.16	< 0.001
Common	6,593 (70.44)	5,869 (89.02)	724 (10.98)		
Poor	941 (10.05)	802 (85.23)	139 (14.77)		
**Education level, years**, ***n*** **(%)**					
0	4,345 (46.42)	3,603 (82.92)	742 (17.08)	χ^2^ = 355.72	< 0.001
1–6	3,147 (33.62)	2,958 (93.99)	189 (6.01)		
≥7	1,868 (19.96)	1,800 (96.36)	68 (3.64)		
**Smoking**, ***n*** **(%)**					
No	7,929 (84.71)	6,999 (88.27)	930 (11.73)	χ^2^ = 60.66	< 0.001
Yes	1,431 (15.29)	1,362 (95.18)	69 (4.82)		
**Drinking**, ***n*** **(%)**					
No	7,975 (85.20)	7,046 (88.35)	929 (11.65)	χ^2^ = 53.83	< 0.001
Yes	1,385 (14.80)	1,315 (94.95)	70 (5.05)		
**Exercise**, ***n*** **(%)**					
No	6,206 (66.30)	5,307 (85.51)	899 (14.49)	χ^2^ = 280.84	< 0.001
Yes	3,154 (33.70)	3,054 (96.83)	100 (3.17)		
**Living arrangements**, ***n*** **(%)**					
Living with household members	7,517 (80.31)	6,680 (88.87)	837 (11.13)	χ^2^ = 112.05	< 0.001
Living alone	1,531 (16.36)	1,447 (94.51)	84 (5.49)		
Living in an institution	312 (3.33)	234 (75.00)	78 (25.00)		
**BMI**, ***n*** **(%)**					
18.5–23.99	4,876 (52.09)	4,371 (89.64)	505 (10.36)	χ^2^ = 193.18	< 0.001
< 18.5	1,470 (15.71)	1,174 (79.86)	296 (20.14)		
24–27.99	2,247 (24.01)	2,108 (93.81)	139 (6.19)		
≥28	767 (8.19)	708 (92.31)	59 (7.69)		
**Hypertension**, ***n*** **(%)**					
No	5,490 (58.65)	4,805 (87.52)	685 (12.48)	χ^2^ = 45.33	< 0.001
Yes	3,870 (41.35)	3,556 (91.89)	314 (8.11)		
**Diabetes**, ***n*** **(%)**					
No	8,420 (89.96)	7,484 (88.88)	936 (11.12)	χ^2^ = 17.28	< 0.001
Yes	940 (10.04)	877 (93.30)	63 (6.70)		
**Heart disease**, ***n*** **(%)**					
No	7,803 (83.37)	6,986 (89.53)	817 (10.47)	χ^2^ = 2.02	0.155
Yes	1,557 (16.63)	1,375 (88.31)	182 (11.69)		
**Dyslipidemia**, ***n*** **(%)**					
No	8,868 (94.74)	7,901 (89.10)	967 (10.90)	χ^2^ = 9.47	0.002
Yes	492 (5.26)	460 (93.50)	32 (6.50)		

χ^2^, Chi-square test; BMI, body mass index.

Additionally, this study included 588 older adults who visited the Thirteenth People's Hospital of Chongqing (Chongqing Geriatric Hospital) between June 2025 and July 2025, with sample information detailed in Supplementary Table S4. Among these, 507 participants (86.22%) were non-demented and 81 (13.78%) had dementia. Among this sample, 54.25% were male and 45.75% were female. The majority of participants (65.65%) were aged 80 years or older. Significant differences in the proportions of participants in the dementia and non-dementia groups were observed across various age, gender, marital status, economic status, education level, smoking status, drinking status, exercise habits, living arrangements, BMI, hypertension, heart disease, and dyslipidemia categories (*P* < 0.05).

### 3.2 Association between plant-based dietary patterns and dementia

To evaluate the associations linking plant-derived eating habits with dementia prevalence, three logistic regression models were formulated. As shown in Table 2, in Model 1, which was unadjusted for any covariates, the scores of PDI, hPDI, and uPDI were significantly associated with dementia (PDI: OR = 0.931, 95% CI = 0.919–0.942; hPDI: OR = 0.937, 95% CI = 0.925–0.948; uPDI: OR = 1.045, 95% CI = 1.035–1.055). In Model 3, after further adjusting for all covariates, these associations remained statistically significant (PDI: OR = 0.964, 95% CI = 0.951–0.977; hPDI: OR = 0.976, 95% CI = 0.963–0.990; uPDI: OR = 1.012, 95% CI = 1.001–1.024). In Model 3, E-values were further calculated to assess the potential influence of unmeasured confounding. The E-values (PDI: 1.55; hPDI: 1.26; uPDI: 1.88) indicated that the influence of unmeasured confounding factors on the association between plant-based diet indices and dementia was small. Overall, from Model 1 to Model 3, the magnitude of these associations decreased slightly as additional covariates were included.

**Table 2:** Association of PDI, hPDI, and uPDI with dementia (*n* = 9,360).

**Model**	**Plant-based dietary patterns**		
	**PDI**	**hPDI**	**uPDI**
Model 1	0.931 (0.919, 0.942)^***^	0.937 (0.925, 0.948)^***^	1.045 (1.035, 1.055)^***^
Model 2	0.959 (0.946, 0.971)^***^	0.971 (0.958, 0.983)^***^	1.011 (1.001, 1.022)^*^
Model 3	0.964 (0.951, 0.977)^***^	0.976 (0.963, 0.990)^***^	1.012 (1.001, 1.024)^*^

PDI, overall plant-based diet index; hPDI, healthful plant-based diet index; uPDI, unhealthful plant-based diet index.

Data are presented as weighted odds ratios (95% confidence intervals).

^*^P < 0.05, ^***^P < 0.001

Model 1: Unadjusted variables.

Model 2: Adjusted for gender, age, residence, marital status, economic status, education level.

Model 3: Further adjusted for smoking, drinking, exercise, living arrangements, BMI, hypertension, diabetes, heart disease, dyslipidemia.

Within the Chongqing validation cohort, we further analyzed associations of PDI, hPDI, and uPDI with dementia prevalence, with detailed results presented in Supplementary Table S5. In Model 3, after adjusting for all covariates, the scores of PDI, hPDI, and uPDI remained significantly associated with dementia (PDI: OR = 0.919, 95% CI = 0.869–0.972; hPDI: OR = 0.943, 95% CI = 0.892–0.998; uPDI: OR = 1.012, 95% CI = 1.000–1.024). The corresponding E-values for Model 3 were 2.01, 1.78, and 1.44, respectively.

### 3.3 Restricted cubic spline analysis

**Figure 2:**
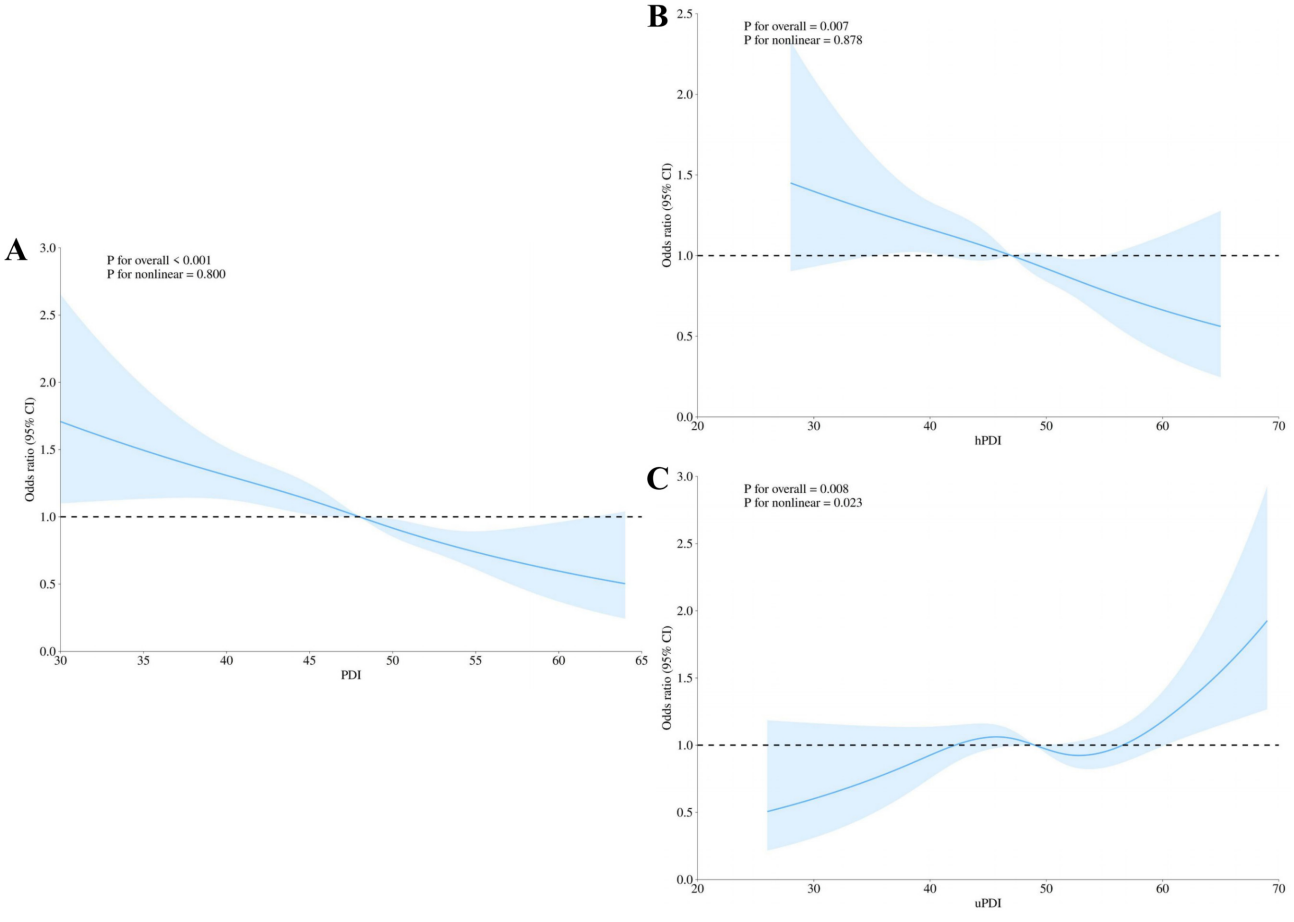
Restricted cubic spline (RCS) analysis for the associations of plant-based diet indices with dementia in the CLHLS main cohort (*N* = 9,360). **(A)** Overall Plant-based Diet Index (PDI); **(B)** Healthy Plant-based Diet Index (hPDI); and **(C)** Unhealthy Plant-based Diet Index (uPDI). Curves and shaded areas represent the odds ratio (OR) and 95% confidence interval (CI), respectively. All analyses were fully adjusted according to Model 3. *P* for overall indicates the *P*-value for the overall association, and *P* for nonlinear tests the nonlinear relationship.

Following comprehensive adjustment for all covariates, restricted cubic spline (RCS) regression modeling was implemented to characterize the dose-response relationships between plant-based diet indices (PDI, hPDI, uPDI) and dementia prevalence. As shown in Figures 2A, B, PDI and hPDI exhibited a significant linear association with dementia (*P* for non-linear > 0.05), while uPDI did not show a significant linear association with dementia (*P* for non-linear < 0.05) (Figure 2C). Furthermore, in the external survey conducted in Chongqing, PDI and hPDI showed a significant linear association with dementia (*P* for non-linear > 0.05), and uPDI did not show a significant linear association with dementia (*P* for non-linear < 0.05) (Supplementary Figure S2).

### 3.4 Subgroup and interaction analysis

**Figure 3:**
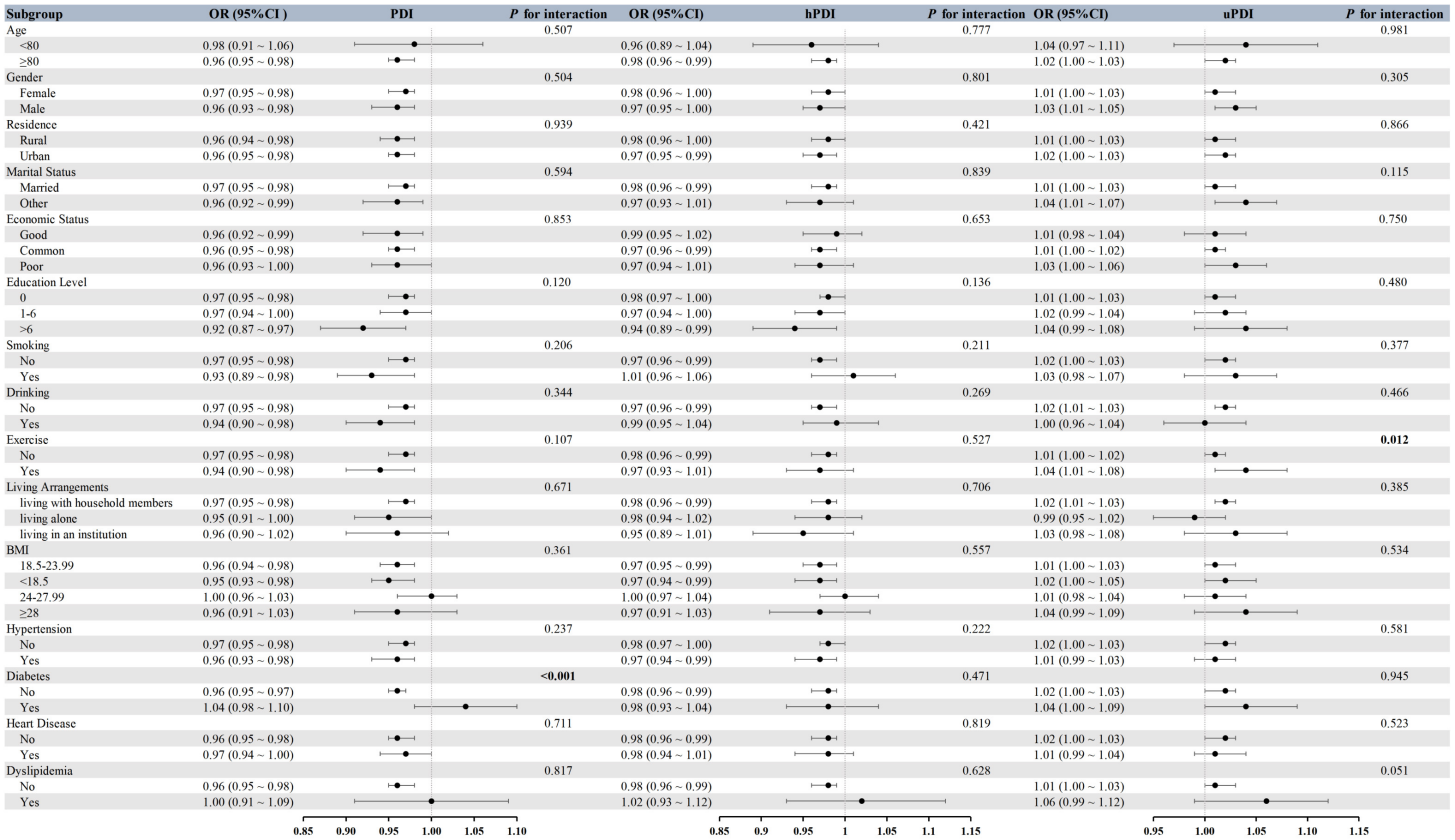
Subgroup and interaction analyses for the associations of Plant-based Diet Indices (PDI, hPDI, and uPDI) with dementia in the CLHLS main cohort (*N* = 9,360). Dots and horizontal lines represent the odds ratio (OR) and its 95% confidence interval (CI), respectively. All subgroup analyses were adjusted according to Model 3, controlling for all covariates except the stratifying variable. *P* for interaction indicates the statistical significance of the interaction between the subgroup variable and the diet indices.

Findings from the subgroup and interaction assessments are displayed in Figure 3. Subgroup analysis indicated that the association between PDI and dementia remained significant across various subgroups, including those aged ≥80 years, different genders, residences, marital statuses, economic statuses, education levels, smoking, drinking, exercise habits, living arrangements, normal and low BMI categories, different hypertension statuses, non-diabetic individuals, and individuals without heart disease. Interaction analysis revealed that diabetes significantly moderated the association between PDI and dementia (*P* for interaction < 0.001).

The association between hPDI and dementia was also significant in subgroups including different genders, urban residents, married individuals, those with common economic status, different education levels, non-smokers, non-drinkers, those living with household members, normal and low BMI categories, different hypertension statuses, and non-diabetic individuals. No significant moderating factors were found in the interaction analysis for hPDI.

The association between uPDI and dementia was significant in subgroups including those aged ≥80 years, different genders, married individuals, those with common economic status, non-smokers, non-drinkers, those who exercise, those living with household members, low BMI categories, non-hypertensive individuals, non-diabetic individuals, and individuals without heart disease. Interaction analysis revealed that exercise significantly moderated the association between uPDI and dementia (*P* for interaction = 0.012).

### 3.5 Sensitivity analysis

For confirming the stability of our principal results, three supplementary sensitivity analyses were performed using the CLHLS dataset. First, we implemented the Multiple Imputation by Chained Equations (MICE) algorithm to address missing data, thereby mitigating potential bias associated with complete-case analysis. Second, we excluded participants with hypertension, diabetes, heart disease, or dyslipidemia, as these chronic disease populations typically have specific dietary restrictions. Association analyses conducted on the two newly obtained datasets consistently aligned with our main study findings (Supplementary Tables S6, S7). In addition, given that dementia generally has a relatively low prevalence, odds ratios derived from logistic regression models may overestimate the strength of associations. Therefore, we conducted a Poisson regression analysis with robust standard errors (Huber-White sandwich estimator) to re-evaluate the associations, as presented in Supplementary Table S8. In the Poisson regression models, both PDI and hPDI showed consistent inverse associations with dementia in the fully adjusted Model 3 (PDI: β = −0.027, 95% CI: −0.037 to −0.017; hPDI: β = −0.018, 95% CI: −0.028 to −0.008). In contrast, uPDI demonstrated a positive association with dementia (uPDI: β = 0.009, 95% CI: 0.000 to 0.018). These findings remained consistent with the main analysis results.

## 4 Discussion

Findings from both datasets in this study consistently demonstrate a statistically significant association between the Healthful Plant-based Diet Index (hPDI) and dementia prevalence, largely consistent with existing international epidemiological evidence (12, 38). Similar patterns were also observed in a Taiwanese cohort study, where adherence to a vegetarian diet was linked to lower dementia occurrence compared with non-vegetarians (39). However, some discrepancies exist in other study results. For instance, one population-based study found no significant association between plant-based diets and dementia incidence (40). In contrast, analyses of UK Biobank data revealed inverse associations between hPDI and dementia incidence, positive associations for uPDI, and null associations for the overall PDI (41). These observed discrepancies may stem from various potential sources, such as differences in genetic predispositions, lifestyle factors, and socioeconomic conditions across distinct geographical populations, all of which can modulate the diet-disease relationship.

The observed associations in this study may be partly explained by several biological properties of plant-derived foods. Healthful plant-based items are rich in bioactive compounds such as vitamins C and E, polyphenols, and flavonoids, which possess strong antioxidant and anti-inflammatory capacities and are considered important in counteracting oxidative stress involved in neurodegenerative processes (42). In addition, these diets provide substantial amounts of dietary fiber, potassium, and vegetable oils, which can help regulate lipid levels, stabilize blood pressure, and improve endothelial function, thereby supporting cerebrovascular health and adequate cerebral perfusion, factors related to a lower prevalence of vascular dementia (43). Dietary fiber also promotes the growth of beneficial gut microbiota and the production of short-chain fatty acids (SCFAs), which may enhance intestinal barrier function, reduce systemic inflammation, and potentially modulate the brain's inflammatory and metabolic milieu via the gut–brain axis, thus contributing to neuroprotection (44, 45).

In the interaction analysis, we observed that both diabetes and physical activity significantly modified the associations between plant-based diet indices and dementia. Diabetes is a complex metabolic condition characterized by cerebral insulin resistance and impaired insulin signaling, which result in reduced neuronal glucose utilization and promote oxidative stress and neuroinflammation (46, 47). In individuals with diabetes, these underlying pathophysiological processes already impose substantial threats to cognitive function. As a result, the associations between healthful plant-based diets and dementia may not be fully evident in this subgroup, since the diet alone is unlikely to counterbalance diabetes-related neurodegenerative alterations. This finding suggests that in older adults with diabetes, dietary patterns should be examined alongside comprehensive disease management, including glycemic control and pharmacological treatment, when exploring their associations with cognitive health.

A considerable body of literature has highlighted the important role of physical activity in relation to dementia and cognitive decline (48, 49). Evidence from a cohort study indicated that higher levels of overall activity, physical activity, or cognitive engagement were associated with lower odds of cognitive impairment (49), while another study suggested that adherence to specific dietary patterns in combination with aerobic and resistance exercise was linked to reduced age-related cognitive decline (50). In line with these findings, the present study also observed that physical activity significantly modified the associations between plant-based dietary indices and dementia. Notably, among participants who engaged in regular exercise, the unhealthful Plant-based Diet Index (uPDI) showed a significant positive association with dementia prevalence, whereas this association was relatively weaker in those without exercise. This suggests that the negative effects of unhealthy plant-based diets might be more pronounced in physically active individuals, potentially offsetting some of the benefits derived from exercise. This could reflect that individuals who exercise are generally more health-conscious, and when they consume unhealthy plant-based foods, the negative impacts of these foods (e.g., inflammation and oxidative stress caused by high sugar, high salt, refined carbohydrates) may become more prominent against a generally “healthier” physiological background. Physical exercise is a recognized intervention for cognitive protection, with mechanisms including enhancing mitochondrial function (51), promoting brain-derived neurotrophic factor expression (52, 53), improving cerebral blood flow, and reducing neuroinflammation (54). The findings of this study suggest that even with active physical exercise, the potential harm to cognitive health from dietary patterns leaning toward unhealthy plant-based foods should not be overlooked, emphasizing the importance of the synergistic action of diet and exercise. This provides more precise recommendations for public health interventions, indicating that promoting a healthy lifestyle should not be a single-dimensional approach but rather a multi-dimensional, synergistic one, especially for physically active individuals, where the healthfulness of their dietary patterns should be particularly emphasized.

## 5 Limitations

Although this investigation provided valuable epidemiological information on the association between plant-based diet and dementia in China's older population through two large and robust datasets, several methodological issues should be noted. First, because of the main reliance on self-report measures, the collected data might be subject to recall errors and social desirability bias. Although previous studies have found the reliability of self- assessed data (55), it is still reasonable to employ more accurate methods to assess this information in future studies. Secondly, the inherent cross-sectional nature of this investigation restricts the ability to establish a causal link between plant-based dietary habits and dementia risk. This should be further evaluated in future cohort studies and intervention trials. Third, in addition to extensive adjustments for covariates, our models were supplemented with E-value analyses. Future investigations may be more comprehensive models including currently unmeasured confounders (e.g., genetic confounding, environmental confounding) into models. Finally, due to limitations in the sampling approach: the external validation cohort included only patients recruited from a single hospital, the dataset may lack adequate representativeness. Therefore, the corresponding findings should be interpreted with caution. Future studies based on larger and more representative samples are needed to further verify these results and provide stronger evidence for future clinical and public health research.

## 6 Conclusion

By employing a nationally representative data set with regional validation data, this study provides new evidence of significant associations of plant-derived dietary patterns with dementia prevalence among older Chinese adults. Restricted cubic spline analysis showed the dose-response relationships of overall and healthful PDI (PDI, hPDI) were associated with dementia. Furthermore, diabetes status and physical activity levels showed significant moderating effects on the associations between plant-based diet and dementia. The identification of modifiable factors provides new leads for intervention of dementia among Chinese older adults. The findings support the formulation of age-specific dietary guidelines that emphasize healthful plant foods while limiting unhealthful variants. From a public-health perspective, the results advocate for risk-stratified, individualized dietary strategies that account for metabolic conditions and physical-activity levels, offering a pragmatic pathway to contribute to understanding the growing dementia burden among older adults in China.

## Data Availability

The data used in this study are from the Chinese Longitudinal Healthy Longevity Survey (CLHLS). The CLHLS datasets are publicly available via the Peking University Open Research Data Platform and can be accessed upon user registration and approval at https://opendata.pku.edu.cn/dataverse/CHADS.
