# Correction: Association between plant-based dietary patterns and dementia among Chinese older adults

**DOI:** 10.3389/fnut.2025.1751473

**Published:** 2026-01-07

**Authors:** Xiaobing Xian, Yue Chen, Yandi Fu, Ziyi Chen, Jie Xiang, Ze Han, Li Zeng, Jiaxia Li, Yuanyuan Wang, Kun Shen

**Affiliations:** 1 The Thirteenth People's Hospital of Chongqing, Chongqing, China; 2 Chongqing Geriatrics Hospital, Chongqing, China; 3 The First Clinical College, Chongqing Medical University, Chongqing, China; 4 School of Pediatric, Chongqing Medical University, Chongqing, China; 5 College of Traditional Chinese Medicine, Chongqing Medical University, Chongqing, China; 6 The Second Clinical College, Chongqing Medical University, Chongqing, China; 7 School of Stomatology, Chongqing Medical University, Chongqing, China; 8 School of Mathematics and Statistics, Chongqing Technology and Business University, Chongqing, China

**Keywords:** plant-based dietary patterns, dementia, older adults, CLHLS, external validation

There was a mistake in Figure 2 as published. Figure 2 in the main text was erroneously replaced with Supplementary Figure 1. The corrected figure 2 and its caption appears below.

**Figure 2:**
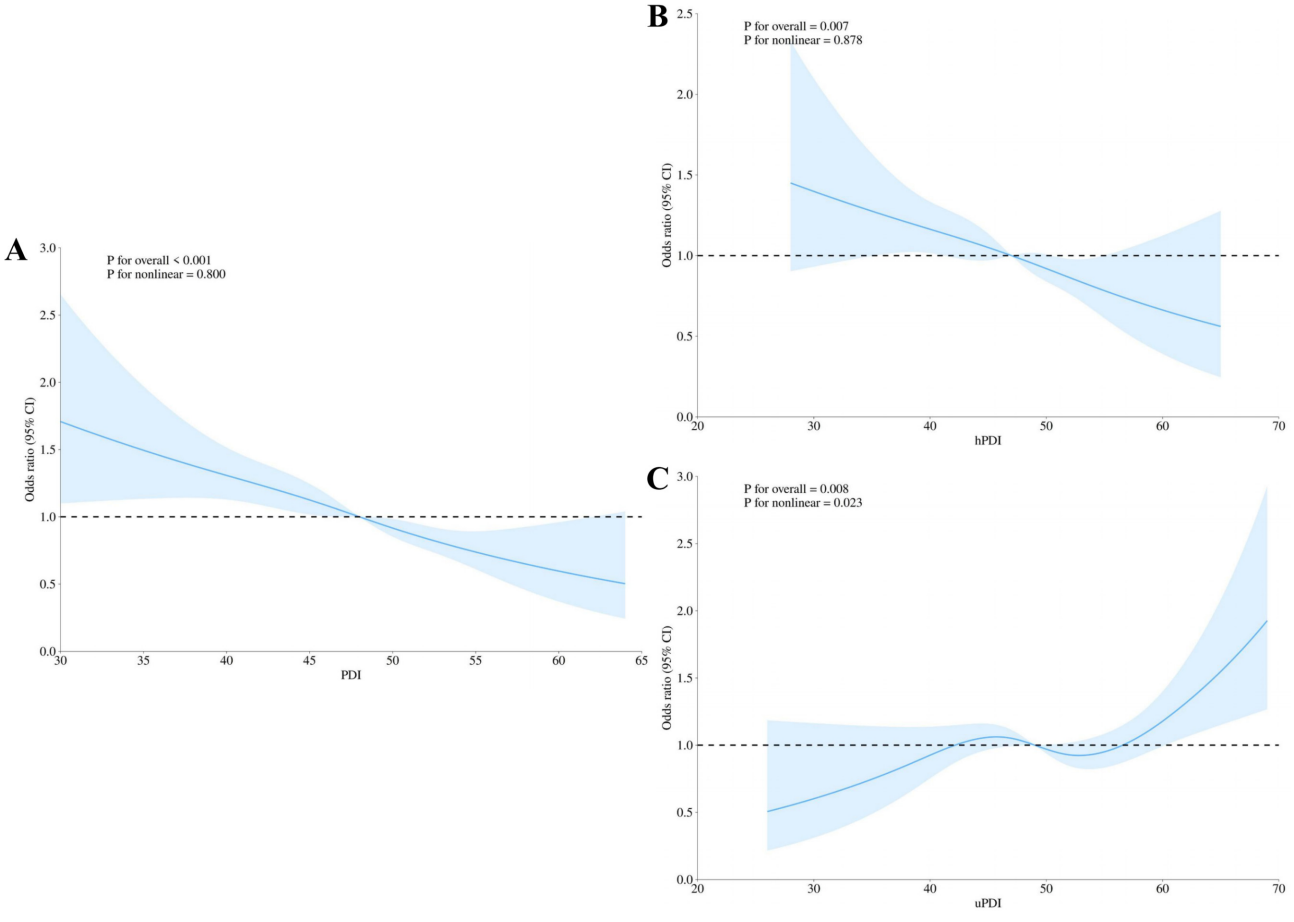
Restricted cubic spline (RCS) analysis for the associations of plant-based diet indices with dementia in the CLHLS main cohort (*N* = 9,360). **(A)** Overall Plant-based Diet Index (PDI); **(B)** Healthy Plant-based Diet Index (hPDI); and **(C)** Unhealthy Plant-based Diet Index (uPDI). Curves and shaded areas represent the odds ratio (OR) and 95% confidence interval (CI), respectively. All analyses were fully adjusted according to Model 3. *P* for overall indicates the *P*-value for the overall association, and *P* for nonlinear tests the nonlinear relationship.

The original version of this article has been updated.

