# Supplementary material for: Association between plant-based dietary patterns and dementia among Chinese older adults
Source: Front Nutr. 2025 Nov 21;12:1669647. doi: 10.3389/fnut.2025.1669647 (PMC12678108; doi:10.3389/fnut.2025.1669647)
Supplement: Supplementary file 1 [file Table_1.DOCX]

***Supplementary Material***

**Table S1. Criteria for classification and assignment of covariates.**

| **Food category** | **Food** | **Frequency** | **PDI** | **hPDI** | **uPDI** |
| --- | --- | --- | --- | --- | --- |
| Plant food | Whole grain | Yes | 5 | 5 | 1 |
|  |  | No | 1 | 1 | 5 |
|  | Vegetable oil | Yes | 5 | 5 | 1 |
|  |  | No | 1 | 1 | 5 |
|  | Fresh fruit | Almost everyday | 5 | 5 | 1 |
|  |  | Quite often | 4 | 4 | 2 |
|  |  | Occasionally | 2 | 2 | 4 |
|  |  | Rarely or never | 1 | 1 | 5 |
|  | Fresh vegetable | Almost everyday | 5 | 5 | 1 |
|  |  | Quite often | 4 | 4 | 2 |
|  |  | Occasionally | 2 | 2 | 4 |
|  |  | Rarely or never | 1 | 1 | 5 |
|  | Legume | Almost everyday | 5 | 5 | 1 |
|  |  | ≥1 time/week | 4 | 4 | 2 |
|  |  | ≥1 time/month | 3 | 3 | 3 |
|  |  | Occasionally | 2 | 2 | 4 |
|  |  | Rarely or never | 1 | 1 | 5 |
|  | Garlic | Almost everyday | 5 | 5 | 1 |
|  |  | ≥1 time/week | 4 | 4 | 2 |
|  |  | ≥1 time/month | 3 | 3 | 3 |
|  |  | Occasionally | 2 | 2 | 4 |
|  |  | Rarely or never | 1 | 1 | 5 |
|  | Nut | Almost everyday | 5 | 5 | 1 |
|  |  | ≥1 time/week | 4 | 4 | 2 |
|  |  | ≥1 time/month | 3 | 3 | 3 |
|  |  | Occasionally | 2 | 2 | 4 |
|  |  | Rarely or never | 1 | 1 | 5 |
|  | Tea | Almost everyday | 5 | 5 | 1 |
|  |  | ≥1 time/week | 4 | 4 | 2 |
|  |  | ≥1 time/month | 3 | 3 | 3 |
|  |  | Occasionally | 2 | 2 | 4 |
|  |  | Rarely or never | 1 | 1 | 5 |
|  | Refined grain | Yes | 5 | 1 | 5 |
|  |  | No | 1 | 5 | 1 |
|  | Sugar | Almost everyday | 5 | 1 | 5 |
|  |  | ≥1 time/week | 4 | 2 | 4 |
|  |  | ≥1 time/month | 3 | 3 | 3 |
|  |  | Occasionally | 2 | 4 | 2 |
|  |  | Rarely or never | 1 | 5 | 1 |
|  | Salt-preserved vegetable | Almost everyday | 5 | 1 | 5 |
|  |  | ≥1 time/week | 4 | 2 | 4 |
|  |  | ≥1 time/month | 3 | 3 | 3 |
|  |  | Occasionally | 2 | 4 | 2 |
|  |  | Rarely or never | 1 | 5 | 1 |
| Animal food | Animal fat | Yes | 1 | 1 | 1 |
|  |  | No | 5 | 5 | 5 |
|  | Meat | Almost everyday | 1 | 1 | 1 |
|  |  | ≥1 time/week | 2 | 2 | 2 |
|  |  | ≥1 time/month | 3 | 3 | 3 |
|  |  | Occasionally | 4 | 4 | 4 |
|  |  | Rarely or never | 5 | 5 | 5 |
|  | Fish | Almost everyday | 1 | 1 | 1 |
|  |  | ≥1 time/week | 2 | 2 | 2 |
|  |  | ≥1 time/month | 3 | 3 | 3 |
|  |  | Occasionally | 4 | 4 | 4 |
|  |  | Rarely or never | 5 | 5 | 5 |
|  | Egg | Almost everyday | 1 | 1 | 1 |
|  |  | ≥1 time/week | 2 | 2 | 2 |
|  |  | ≥1 time/month | 3 | 3 | 3 |
|  |  | Occasionally | 4 | 4 | 4 |
|  |  | Rarely or never | 5 | 5 | 5 |
|  | Dairy products | Almost everyday | 1 | 1 | 1 |
|  |  | ≥1 time/week | 2 | 2 | 2 |
|  |  | ≥1 time/month | 3 | 3 | 3 |
|  |  | Occasionally | 4 | 4 | 4 |
|  |  | Rarely or never | 5 | 5 | 5 |
| Note: PDI: Overall Plant-Based Diet Index; hPDI: Healthful Plant-Based Diet Index; uPDI: Unhealthful Plant-Based Diet Index. | | | | | |

**Table S2. Questions about the CMMSE scale.**

| **Classification** | |
| --- | --- |
| General competence  (12 marks) | Question 1: What time is it, morning, noon, afternoon, or evening (correct=1, incorrect or unable to answer=0) |
|  | Question 2: What month is it (correct=1, incorrect or unable to answer=0) |
|  | Question 3: When is the Mid-Autumn Festival (correct=1, incorrect or unable to answer=0) |
|  | Question 4: What season is it (correct=1, incorrect or unable to answer=0) |
|  | Question 5: Name the district or commune you live (correct=1, incorrect or unable to answer=0) |
|  | Question 6: Name the things that can be eaten (1 mark for 1 correct answer, 7 marks for 7 or more answers) |
| Reactivity  (3 marks) | Question 1: Name the “table” correctly (correct=1, incorrect or unable to answer=0) |
|  | Question 2: Name the “apple” correctly (correct=1, incorrect or unable to answer=0) |
|  | Question 3: Name the “clothes” correctly (correct=1, incorrect or unable to answer=0) |
| Attention and numeracy  (6 marks) | Question 1: 20–3=? (correct=1, incorrect or unable to answer=0) |
|  | Question 2: 20–3-3=? (correct=1, incorrect or unable to answer=0) |
|  | Question 3: 20–3-3-3=? (correct=1, incorrect or unable to answer=0) |
|  | Question 4: 20–3–3-3-3-3=? (correct=1, incorrect or unable to answer=0) |
|  | Question 5: 20–3–3-3-3-3-3=? (correct=1, incorrect or unable to answer=0) |
|  | Question 6: Draw the figure on the card (correct=1, incorrect or incomplete=0) |
| Recollection skills  (3 marks) | Question: Repeat “table, apple, clothes” as remembered in the “responsiveness section”(1 mark for 1 correct answer; do not count the order of answers) |
| Language, comprehension, and self coordination skills  (6 marks) | Question 1: Name the object the investigator is pointing to as “pen” (correct=1, incorrect or unable to answer=0) |
|  | Question 2: Name the “watch” to which the investigator is referring (correct=1, incorrect or unable to answer=0) |
|  | Question 3: Repeat the assigned sentence from the investigator (correct=1, incorrect or unable to answer=0) |
|  | Question 4: Ask the respondent to hold the paper in their right hand (correct=1, incorrect or unable to complete=0) |
|  | Question 5: Ask the respondent to fold the paper in half (correct=1, incorrect or unable to complete=0) |
|  | Question 6: Ask the respondent to place the paper on the floor (correct=1, incorrect or unable to complete=0) |

**Table S3. Measurements and classification criteria of covariates.**

| **Variables** | **Questionnaire Items** | **Classification Criteria** |
| --- | --- | --- |
| Age | What’s your current age? | <80=0; ≥80=1 |
| Gender | What’s your gender? | Female=0; Male=1 |
| Residence | Was your address in the rural or urban? | Rural=0; Urban=1 |
| Marital status | What is your marital status? | Unmarried=0; Married=1 |
| Economic status | How do you rate your economic status? | Good=0; Common=1; Poor=2 |
| Education level | How many years of education did you have? | 0=0; 0~6=1; >6=2 |
| Smoking | Smoke or not at present? | No=0; Yes=1 |
| Drinking | Drink or not at present? | No=0; Yes=1 |
| Exercise | Exercise or not at present? | No=0; Yes=1 |
| Living arrangements | What are your current living arrangements? | Living with household members=0;  Living alone=1;  Living in an institution=2 |
| Hypertension | Have you been diagnosed with hypertension by a doctor? | No=0; Yes=1 |
| Diabetes | Have you been diagnosed with diabetes by a doctor? | No=0; Yes=1 |
| Heart disease | Have you been diagnosed with heart disease by a doctor? | No=0; Yes=1 |
| Dyslipidemia | Have you been diagnosed with dyslipidemia by a doctor? | No=0; Yes=1 |
| BMI | How much do you weight; What’s your height? | 18.5~23.99=0; <18.5=1;  24~27.99=2; ≥28=3 |
| Note: BMI: Body Mass Index= Weight/The square of the height (1). | | |

**Table S4. Characteristics of external survey data at baseline by dementia.**

| **Variables** | **Total**  **(n = 588)** | **Without dementia**  **(n = 507)** | **Dementia**  **(n = 81)** | **Statistic** | ***P*** |
| --- | --- | --- | --- | --- | --- |
|  |  |  |  |  |  |
| Age, n (%) |  |  |  | χ²=42.35 | <0.001 |
| <80 | 202 (34.35) | 200 (99.01) | 2 (0.99) |  |  |
| ≥80 | 386 (65.65) | 307 (79.53) | 79 (20.47) |  |  |
| Gender, n (%) |  |  |  | χ²=8.39 | 0.004 |
| Female | 319 (54.25) | 263 (82.45) | 56 (17.55) |  |  |
| Male | 269 (45.75) | 244 (90.71) | 25 (9.29) |  |  |
| Residence, n (%) |  |  |  | χ²=1.66 | 0.197 |
| Rural | 189 (32.14) | 168 (88.89) | 21 (11.11) |  |  |
| Urban | 399 (67.86) | 339 (84.96) | 60 (15.04) |  |  |
| Marital status, n (%) |  |  |  | χ²=48.20 | <0.001 |
| Married | 328 (55.78) | 254 (77.44) | 74 (22.56) |  |  |
| Other | 260 (44.22) | 253 (97.31) | 7 (2.69) |  |  |
| Economic status, n (%) |  |  |  | χ²=8.62 | 0.013 |
| Good | 109 (18.54) | 103 (94.50) | 6 (5.50) |  |  |
| Common | 441 (75.00) | 370 (83.90) | 71 (16.10) |  |  |
| Poor | 38 (6.46) | 34 (89.47) | 4 (10.53) |  |  |
| Education level, years, n (%) |  |  |  | χ²=16.11 | <0.001 |
| 0 | 254 (43.20) | 204 (80.31) | 50 (19.69) |  |  |
| 1~6 | 190 (32.31) | 167 (87.89) | 23 (12.11) |  |  |
| ≥7 | 144 (24.49) | 136 (94.44) | 8 (5.56) |  |  |
| Smoking, n (%) |  |  |  | χ²=7.10 | 0.008 |
| No | 512 (87.07) | 434 (84.77) | 78 (15.23) |  |  |
| Yes | 76 (12.93) | 73 (96.05) | 3 (3.95) |  |  |
| Drinking, n (%) |  |  |  | χ²=7.24 | 0.007 |
| No | 501 (85.20) | 424 (84.63) | 77 (15.37) |  |  |
| Yes | 87 (14.80) | 83 (95.40) | 4 (4.60) |  |  |
| Exercise, n (%) |  |  |  | χ²=40.96 | <0.001 |
| No | 363 (61.73) | 287 (79.06) | 76 (20.94) |  |  |
| Yes | 225 (38.27) | 220 (97.78) | 5 (2.22) |  |  |
| Living arrangements, n (%) |  |  |  | χ²=39.70 | <0.001 |
| Living with household members | 447 (76.02) | 392 (87.70) | 55 (12.30) |  |  |
| Living alone | 113 (19.22) | 102 (90.27) | 11 (9.73) |  |  |
| Living in an institution | 28 (4.76) | 13 (46.43) | 15 (53.57) |  |  |
| BMI, n (%) |  |  |  | χ²=21.90 | <0.001 |
| 18.5~23.99 | 305 (51.87) | 265 (86.89) | 40 (13.11) |  |  |
| <18.5 | 81 (13.78) | 58 (71.60) | 23 (28.40) |  |  |
| 24~27.99 | 154 (26.19) | 144 (93.51) | 10 (6.49) |  |  |
| ≥28 | 48 (8.16) | 40 (83.33) | 8 (16.67) |  |  |
| Hypertension, n (%) |  |  |  | χ²=4.87 | 0.027 |
| No | 296 (50.34) | 246 (83.11) | 50 (16.89) |  |  |
| Yes | 292 (49.66) | 261 (89.38) | 31 (10.62) |  |  |
| Diabetes, n (%) |  |  |  | χ²=2.41 | 0.120 |
| No | 513 (87.24) | 438 (85.38) | 75 (14.62) |  |  |
| Yes | 75 (12.76) | 69 (92.00) | 6 (8.00) |  |  |
| Heart disease, n (%) |  |  |  | χ²=5.66 | 0.017 |
| No | 459 (78.06) | 404 (88.02) | 55 (11.98) |  |  |
| Yes | 129 (21.94) | 103 (79.84) | 26 (20.16) |  |  |
| Dyslipidemia, n (%) |  |  |  | χ²=4.78 | 0.029 |
| No | 553 (94.05) | 472 (85.35) | 81 (14.65) |  |  |
| Yes | 35 (5.95) | 35 (100.00) | 0 (0.00) |  |  |
| Note: BMI: Body Mass Index; χ²: Chi-square test. | | | | | |

**Table S5. Association of PDI , hPDI , and uPDI with dementia of external survey data (n = 588).**

| **Model** | **Plant-based dietary patterns** | | |
| --- | --- | --- | --- |
|  | **PDI** | **hPDI** | **uPDI** |
| Model 1 | 0.893 (0.853, 0.934)*** | 0.908 (0.866,0.952)*** | 1.036 (1.003,1.070)* |
| Model 2 | 0.912 (0.868,0.959)*** | 0.930 (0.885,0.977)** | 1.009 (0.970,1.049) |
| Model 3 | 0.919 (0.869,0.972)** | 0.943 (0.892,0.998)** | 1.012 (1.000,1.024)* |
| Note: PDI: Overall Plant-Based Diet Index; hPDI: Healthful Plant-Based Diet Index; uPDI: Unhealthful Plant-Based Diet Index.  Data are presented as weighted odds ratios (95% confidence intervals).  * :*P* < 0.05, ** :*P* < 0.01, *** :*P* < 0.001  Model 1: Unadjusted variables.  Model 2: Adjusted for gender, age, residence, marital status, economic status, education level.  Model 3: Further adjusted for smoking, drinking, exercise, living arrangements, BMI, hypertension, diabetes, heart disease, dyslipidemia. | | | |

**Table S6. Association between PDI, hPDI, and uPDI and dementia after multiple imputation of missing data (n = 11,599).**

| **Model** | **Plant-based dietary patterns** | | |
| --- | --- | --- | --- |
|  | **PDI** | **hPDI** | **uPDI** |
| Model 1 | 0.938 (0.928 , 0.949)*** | 0.940 (0.929 , 0.951)*** | 1.043 (1.034 , 1.052)*** |
| Model 2 | 0.974 (0.962 , 0.986)*** | 0.982 (0.970 , 0.995)** | 1.008 (0.997 , 1.019) |
| Model 3 | 0.976 (0.964 , 0.989)*** | 0.984 (0.971 , 0.997)*** | 1.011 (1.000 , 1.022) |
| Note: PDI: Overall Plant-Based Diet Index; hPDI: Healthful Plant-Based Diet Index; uPDI: Unhealthful Plant-Based Diet Index.  Data are presented as weighted odds ratios (95% confidence intervals).  *: *P* < 0.05, ** :P < 0.01, ***: *P* < 0.001  Model 1: Unadjusted variables.  Model 2: Adjusted for gender, age, residence, marital status, economic status, education level.  Model 3: Further adjusted for smoking, drinking, exercise, living arrangements, BMI, hypertension, diabetes, heart disease, dyslipidemia. | | | |

**Table S7. Association between PDI, hPDI, and uPDI and dementia after excluding individuals with chronic diseases (n = 4,623).**

| **Model** | **Plant-based dietary patterns** | | |
| --- | --- | --- | --- |
|  | **PDI** | **hPDI** | **uPDI** |
| Model 1 | 0.940 (0.925 , 0.955)*** | 0.950 (0.935 , 0.965)*** | 1.046 (1.032 , 1.060)*** |
| Model 2 | 0.976 (0.959 , 0.994)** | 0.973(0.957 , 0.989)*** | 1.015 (1.001 , 1.030)* |
| Model 3 | 0.967 (0.951 , 0.984)*** | 0.983 (0.966 , 0.999)* | 1.015 (1.000 , 1.031) |
| Note: PDI: Overall Plant-Based Diet Index; hPDI: Healthful Plant-Based Diet Index; uPDI: Unhealthful Plant-Based Diet Index.  Data are presented as weighted odds ratios (95% confidence intervals).  *: *P* < 0.05, ** :*P* < 0.01, ***: *P* < 0.001  Model 1: Unadjusted variables.  Model 2: Adjusted for gender, age, residence, marital status, economic status, education level.  Model 3: Further adjusted for smoking, drinking, exercise, living arrangements, BMI. | | | |

**Table S8. Poisson regression with robust standard errors: the associations between PDI, hPDI, uPDI and dementia (n = 9,360).**

| **Model** | **Plant-based dietary patterns** | | |
| --- | --- | --- | --- |
|  | **PDI** | **hPDI** | **uPDI** |
| Model 1 | −0.063 (−0.073 , −0.053)*** | −0.058 (−0.068 , −0.047)*** | 0.039 (0.031 , 0.047)*** |
| Model 2 | −0.034 (−0.043 , −0.024)*** | −0.024 (−0.034 , −0.014)*** | 0.009 (0.000 , 0.018)* |
| Model 3 | −0.027 (−0.037 , −0.017)*** | −0.018 (−0.028 , −0.008)*** | 0.009 (0.000 , 0.018)* |
| Note: PDI: Overall Plant-Based Diet Index; hPDI: Healthful Plant-Based Diet Index; uPDI: Unhealthful Plant-Based Diet Index.  * :*P* < 0.05 , ***: *P* < 0.001  Model 1: Unadjusted variables.  Model 2: Adjusted for gender, age, residence, marital status, economic status, education level.  Model 3: Further adjusted for smoking, drinking, exercise, living arrangements, BMI, hypertension, diabetes, heart disease, dyslipidemia. | | | |

**
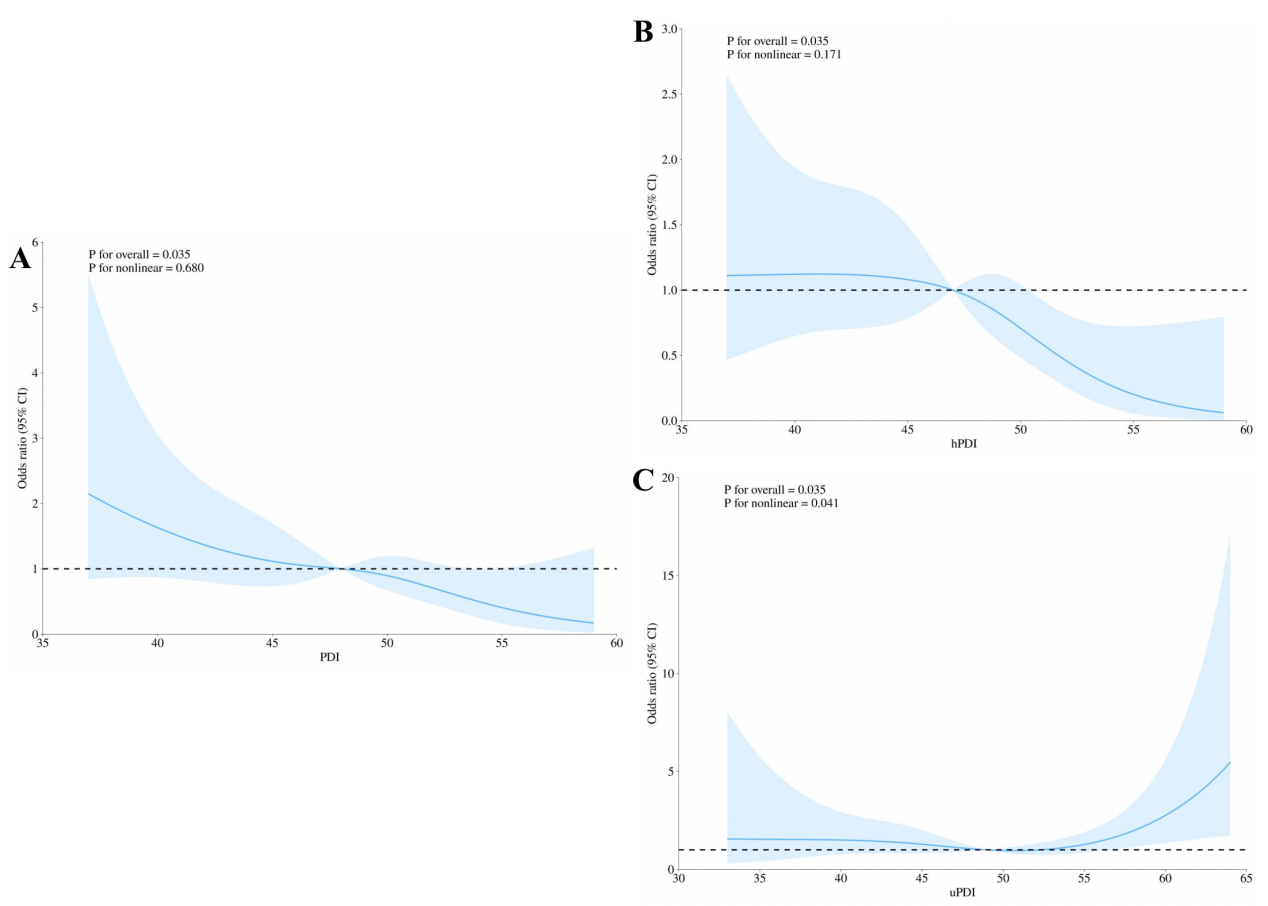
**

**Fig.S1 Restricted cubic spline for testing the hypothesis of nonlinear correlation between PDI, hPDI, and uPDI and dementia of external survey data (n = 588).**

Figure.S1 Restricted cubic spline (RCS) analysis for the associations of Plant-based Diet Indices (PDI, hPDI, and uPDI) with dementia in the CLHLS nested case-control study.(A) Overall Plant-based Diet Index (PDI); (B) Healthy Plant-based Diet Index (hPDI); (C) Unhealthy Plant-based Diet Index (uPDI).Curves and shaded areas represent the odds ratio (OR) and 95% confidence interval (CI), respectively. All analyses were fully adjusted according to Model 3. *P* for overall indicates the *P*-value for the overall association, and *P* for nonlinear tests the nonlinear relationship.

**References**

1. Associations of body mass index, waist circumference and waist-to-height ratio with cognitive impairment among Chinese older adults: based on the CLHLS. J Affect Disord. 2021 Dec 1;295:463–70.
